# Melatonin and Microglia

**DOI:** 10.3390/ijms22158296

**Published:** 2021-08-02

**Authors:** Rüdiger Hardeland

**Affiliations:** Johann Friedrich Blumenbach Institute of Zoology and Anthropology, University of Göttingen, 37073 Göttingen, Germany; rhardel@gwdg.de; Tel.: +49-551-782270

**Keywords:** CNS, inflammation, melatonin, microglia, microRNAs, sirtuins

## Abstract

Melatonin interacts in multiple ways with microglia, both directly and, via routes of crosstalk with astrocytes and neurons, indirectly. These effects of melatonin are of relevance in terms of antioxidative protection, not only concerning free-radical detoxification, but also in prevention of processes that cause, promote, or propagate oxidative stress and neurodegeneration, such as overexcitation, toxicological insults, viral and bacterial infections, and sterile inflammation of different grades. The immunological interplay in the CNS, with microglia playing a central role, is of high complexity and includes signaling toward endothelial cells and other leukocytes by cytokines, chemokines, nitric oxide, and eikosanoids. Melatonin interferes with these processes in multiple signaling routes and steps. In addition to canonical signal transduction by MT_1_ and MT_2_ melatonin receptors, secondary and tertiary signaling is of relevance and has to be considered, e.g., via the upregulation of sirtuins and the modulation of pro- and anti-inflammatory microRNAs. Many details concerning the modulation of macrophage functionality by melatonin are obviously also applicable to microglial cells. Of particular interest is the polarization toward M2 subtypes instead of M1, i.e., in favor of being anti-inflammatory at the expense of proinflammatory activities, which is well-documented in macrophages but also applies to microglia.

## 1. Introduction

The multiple and partially divergent activities of microglia are of crucial importance to the initiation, course, and termination of inflammation in the central nervous system. Crosstalk between microglia and other cells, especially but not exclusively astrocytes and neurons, are also involved in these processes. Melatonin has been shown to act in protective and mostly anti-inflammatory ways under various conditions of high- and low-grade brain inflammation, including age-related impairments in the course of immunosenescence, neurodegenerative disorders, and bacterial and viral infections as well as in processes caused by ischemia/reperfusion or brain trauma [1,2,3,4]. These actions comprise various types of signaling, including the suppression of inflammatory signals, the enhancement of antioxidant and antinitrosant metabolism, mitochondrial protection, and the scavenging of reactive oxygen and nitrogen species (ROS, RNS). An additional and presumably highly important aspect of melatonin’s anti-inflammatory potential has been observed in macrophages, i.e., cells that are functionally and developmentally related to microglia. Melatonin was shown to strongly influence their polarization in favor of the anti-inflammatory type M2 at the expense of the proinflammatory type M1 [5].

**Figure 1:**
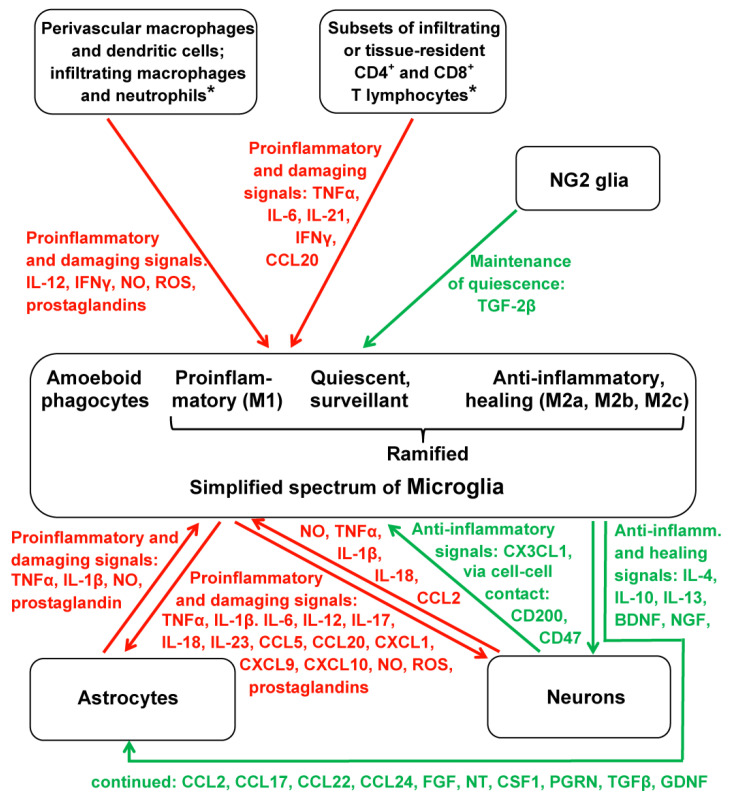
Overview of microglial subtypes and their pro- and anti-inflammatory cellular environment. Proinflammatory interactions and factors are depicted in red, anti-inflammatory interactions and factors are depicted in green. Asterisks (*) indicate a higher complexity of signaling and, in the case of T lymphocytes, a higher diversity of subtypes. Generally, the complexity of interactions is even higher and also concerns differences between various diseases, but the figure had to be kept within an intelligible frame. Damaging factors caused by dying cells at sites of injury have been omitted; this would especially play a role in the focal activation that causes the transformation of ramified microglial cells into amoeboid phagocytes. Additional signaling via microRNAs and other constituents of exosomes has been also omitted. Abbreviations: BDNF, brain-derived neurotrophic factor; CCL, CXCL, and CX3CL refer to the respective chemokine families; CD, cluster of differentiation; CSF1, colony-stimulating factor-1; FGF, fibroblast growth factor; GDNF, glia-derived neurotrophic factor; IFNγ, interferon-γ; IL, interleukin; NG2 glia, neural-glial-2 expressing cells; NGF, nerve growth factor; NO, nitric oxide; NT, neurotrophin (several subforms); PGRN, progranulin; ROS, reactive oxygen species; TNFα, tumor necrosis factor-α; TGFβ, transforming growth factor-β.

Such a mode of polarization in functional differentiation also exists in microglia, although it does not seem to be fully identical to the one observed in macrophages. The differences in functionality can be traced by determining surface and expression markers, which, however, also reveals the necessity of discriminating M2 subtypes [6] that are also known from macrophages [7]. In macrophages, the subtype M2a is concomitantly active with M1 and may serve to prevent overshooting M1 activity. M2b is typically active in the subsequent proliferative phase of wound healing after injury, whereas M2c is associated with a later phase of remodeling or regeneration [7]. In microglia, the exact relationship to specific phases remains to be further investigated under the respective inflammatory conditions. Nevertheless, the distinction between the M2 subtypes in microglia is established [6,8]. It should also be briefly mentioned that a further microglia polarization type, M3, has also been discussed, which was assumed to be activated by MCSF (macrophage colony-stimulating factor) and IL-34 and was identified by the receptor SCF-1R as a specific marker [6]. Again, this conclusion would require further support. Another problem concerning the identification of microglia subtypes under conditions of brain injury is related to the invasion of other leukocytes. In particular, blood monocytes migrate to the injured site, and they differentiate into macrophages that have been reported to be poorly distinguishable from activated microglia in terms of both morphology and the expression of the majority of markers [7,9]. However, CD44 and CD169 have been reported to be macrophage-specific markers absent in microglia, whereas Siglec-H was listed as a microglia-specific marker in mice [9]. Moreover, a subset of Iba-1^+^, CD68^+^ expressing microglia with amoeboid morphology was shown to be distinguished from infiltrated macrophages by the presence of TMEM119 (transmembrane protein 119), which may serve as a reliable microglial marker absent in macrophages [10]. However, TMEM119^+^ cells were reported to not consistently express M1 and M2 markers [11]. Cells originally thought to represent amoeboid microglial cells (AMC) in the developing brain have been reported to be, in fact, active macrophages [11]. Finally, the biology of microglia requires the consideration of a further glia type, the NG2 glia, a term coined according to the presence of nerve/glial antigen 2 (=Neural-Glial 2). These cells, also known as polydendrocytes, which were previously thought to only represent oligodendrocyte precursors, are actually regarded as a fourth category of glia [12,13], with specific functions in the regulation of microglia [14,15,16]. Generally, the functions of microglia cannot be sufficiently understood without considering their interactions with other cell types. Even seemingly quiescent microglial cells interact with neurons, astrocytes, other leukocytes, and vascular cells through filipodial contacts, thereby fulfilling a surveillance function of the microenvironment [9]. Disturbances detected by the microglia can lead to their activation, which can also be initiated by functional changes in the contacted cells. An overview of the interactions between the various cell types and the most important molecules of intercellular signaling is presented in Figure 1. This figure includes the proinflammatory and anti-inflammatory signal molecules released by M1 and M2 microglia, respectively. Moreover, M1 is characterized by the expression of iNOS and the surface markers CD14, CD16, CD32, CD40, and MHC class II, whereas in M2, Arg1, CD163, and CD206 are expressed [9].

When comparing the roles of microglia over the decades, it becomes evident that earlier studies regarded these cells as comparably uniform, mainly differing in their states of quiescence or activation. The roles of actively counteracting inflammatory responses remained largely unconsidered for quite some time. This view was also evident in the earlier interpretation of markers that were only used for categorizing cells as quiescent or activated regardless of whether they were of the proinflammatory M1 type or one of the anti-inflammatory M2 subtypes. Some of the markers were, in fact, unsuitable for discriminating the opposite roles in inflammation, such as Iba-1 and CD68, which are expressed in both M1 and M2 microglia [6,7]. With regard to melatonin literature, the new insights concerning microglia subtypology have only poorly entered a desired state of consideration, although the anti-inflammatory role of melatonin has meanwhile become a hot topic in this field. It is the aim of this article to not only summarize the actual knowledge of melatonin effects in microglia but also to direct the readers’ attention to the requirements of further melatonin research on the basis of microglial diversity and interactive complexity.

## 2. Melatonin Suppresses Proinflammatory and Favors Anti-Inflammatory Signaling

### 2.1. Effects in Microglia on the Background of Known Actions in Macrophages and Tissues

The mechanisms that control inflammation are of utmost importance to numerous pathologies [2,3,4]. They are also related to the counteraction of oxidative and nitrosative stress as well as to mitochondrial function and integrity, i.e., fields that have been regarded as being central to melatonin’s value in maintaining and promoting health. The suppression of proinflammatory signals by melatonin has been repeatedly documented. Respective findings mainly concern the downregulation of iNOS (inducible nitric oxide synthase) [16,17,18,19] and COX-2 (cyclooxygenase-2) [19,20]; the suppression of inflammasome activation, especially that of NLRP3; the antagonism of NF-κB vs. Nrf2; and the reduced release of proinflammatory cytokines, as summarized in pertinent reviews [2,4,21,22,23]. Various additional effects concerning the up- or downregulation of other factors and signaling pathways have also been included in these articles. However, the majority of the earlier findings did not specifically refer to microglia.

Meanwhile, a substantial body of evidence has accumulated concerning the actions of melatonin in microglia. Basically, most of these findings are in good agreement with the earlier data although the aspect of polarization has not yet been generally considered. The suppression of iNOS by melatonin has now been repeatedly confirmed in microglia [24,25,26,27]. This effect can be concluded to concern M1 cell function since iNOS is regarded as a marker of this polarization phenotype [9,28,29,30]. The downregulation of COX-2, originally described in the macrophages [19,20], has meanwhile also been confirmed in microglia [31]. Whether or not this may be associated with changes in polarization would depend on the details of the respective studies, especially concerning the duration of exposure. This may especially apply to a study on acute spinal cord injury, in which increases in M2 markers were also reported, though without strict discrimination between microglia and macrophages [27]. However, some uncertainties remain when immortalized cell lines have been used, such as HAPI [24] or BV2 cells [26]. Such cell lines, especially the aggressively proliferating HAPI cells [24], may have lost the capability of becoming reprogrammed. Maintenance of the M1 type is known from immortalized macrophages, a property that seems to have contributed to earlier assumptions that melatonin may primarily act in a proinflammatory way [4].

### 2.2. Melatonin and Inflammasomes

Suppression of the formation and activity of the NLRP3 inflammasome caused by melatonin was observed after LPS treatment in natural microglia in vivo [32] and in BV2 cells [33] as well as in an inflammation model of intracerebral hemorrhage in which BV2 cells were treated with thrombin [34]. In the latter study, conditioned medium from thrombin-treated BV2 cells was also shown to induce apoptosis in HT22 cells, an effect suppressed by the addition of melatonin. Similar protection was obtained with the antioxidant *N*-acetylcysteine, which may indicate a role of ROS scavenging [34]. The prevention of microglial inflammasome activation may be largely explained by the downregulation of TLR4 (toll-like receptor 4) [35,36,37,38] and its adapter protein MyD88 (myeloid differentiation factor 88) [36], effects that have been observed in very different models and under various conditions. However, reduced TLR4-dependent signaling may also occur upon the prevention of HMGB1 (high mobility group box 1) release, an alarmin that activates TLR4, as reported for LPS-treated BV2 microglial cells [39]. Downstream signaling that is inhibited by melatonin can concern different pathway branches, in particular, the phosphorylation of Akt, mTOR [37,40], and the stress kinases JNK and p38 [37,40]; the prevention of NADPH oxidase activation/assembly [38,40,41]; and the prevention of caspase-3 cleavage [35,36,42]. As a common feature that is observed throughout practically all pertinent studies, the suppression of NF-κB activation, which was often accompanied by Nrf2 upregulation, reflects the anti-inflammatory and antioxidant actions of melatonin in microglia [26,32,36,38,39,43,44,45,46,47,48]. Conversely, the suppression of melatonin secretion by sleep deprivation enhanced microglia activation, which was evident from proinflammatory traits such as NF-κB activation, JNK phosphorylation, the upregulation of NADPH oxidases 1, 2, and 4, and the release of proinflammatory cytokines [49].

### 2.3. Melatonin on Microglial Cytokines and Chemokines

A rather substantial body of evidence exists for the suppression of proinflammatory cytokines by melatonin in microglia. While most studies have only considered IL-1β and TNFα, additional data have been published for IL-6 and IL-18 [26,27,32,33,34,41,42,50,51,52,53,54,55]. Although many other interleukins would also be of interest, researchers have mostly confined their measurements to the aforementioned factors, which already give a clear picture of inflammation induction. Further proinflammatory cytokines influenced by melatonin, without specific experimental reference to microglia, have been summarized elsewhere [2,4]. Notably, all of this information on the anti-inflammatory actions of melatonin in microglia as well as in studies in which the precise sources of the released cytokines had not been determined was obtained in highly different models using diverse experimental settings. This clearly indicates that the inflammation-alleviating role of melatonin is not conditional but rather reflects a fundamental property.

Although a substantial body of data exists on the upregulation of anti-inflammatory M2-typic cytokines by melatonin without the identification of microglia as their source [2,4], respective information specifically related to microglia is still rather scarce. Within this limited field, the upregulation of IL-10, IL-19, and TGFβ have been reported [53], but data on IL-4, IL-13, and various other anti-inflammatory factors are widely missing. The upregulation of BDNF (brain-derived neurotrophic factor) in microglia by melatonin [42,48] may be taken as a hint for a melatonin-directed M2 function that exceeds the suppression of inflammation that concerns proliferation and remodeling phases after injury, as typical for the M2b and M2c subtypes.

Along with the secretion of inflammation-promoting cytokines, various chemokines are also released to attract other leukocytes that infiltrate the site of injury. As an anti-inflammatory agent, melatonin should attenuate this process. Existing data are partially compatible with this assumption. However, the microglia-specific information is, again, rather limited. The downregulation of CCL5 and CCL9 were reported in conjunction with other anti-inflammatory actions [44], but the same study also reported the suppression of CCL2 release, i.e., the release of a chemokine that is usually related to M2 function [9]. In this investigation, the question remains as to whether this atypical response may be a consequence of using the immortalized cell line BV2, which may combine M1 and M2 properties. Another study demonstrated the prevention of a pathologic downregulation of CX3CL1 by melatonin [56].

### 2.4. A Divergent Observation in Retina—Role of RhoA/ROCK Signaling

An article on retinal microglia and/or macrophages concerning a murine model of wet macular degeneration reported that melatonin upregulates CCL3 and CCL5 along with producing an increase of iNOS expression and TNFα release [57]. These findings are opposite to what is normally found and imply favored M1 function at the expense of M2. This aspect of the actions of melatonin may require further clarification, specifically with regard to other tissues, especially those with a high degree of vascularization, including kidney, lung, and heart tissues. The interpretation by authors has been that melatonin shifts in the retina, macrophage/microglia polarization from the pro-angiogenic M2 to the anti-angiogenic M1 phenotype. This was in accordance with the observed inhibition of choroidal neovascularization, a hallmark of advanced wet age-related macular degeneration [57]. These findings go beyond the topic of microglia but shall be briefly addressed because of their potentially broader importance. The re-programming from M2 to M1, which would be equally relevant to macrophages, has been attributed to the inhibition of RhoA/ROCK signaling [57]. This pathway, which is associated with vascular functions, concerns the role of the small GTP-binding protein (“monomeric G protein”) RhoA and its targets, the Rho kinases (ROCK1 and ROCK2). This pathway is activated by various molecules, such as angiotensin II, endothelin-1 (ET-1), thrombin, and several other factors released by M2 microglia [9], e.g., TGFβ and FGF (fibroblast growth factor) [58]. Under both physiological and medical aspects, it would be of utmost importance to find out whether melatonin is more generally capable of suppressing RhoA/ROCK signaling in other tissues, including brain tissue, and in other cell types. However, the findings on polarization obtained in the retina [57] do not yet allow for the generalization of conclusions on responses of retinal microglia to melatonin. In another model of oxygen-induced murine retinopathy, the activation of microglia and the elevation of inflammation mediators were suppressed by melatonin, which indicates activities of M2 rather than M1, but the outcome concerning the inhibition of neovascularization was, surprisingly, rather similar [59].

### 2.5. Prevalent Observations and Aspects of Melatonergic Signaling

Despite the deviating effect in retinal microglia that may reflect an unexpected change in polarization towards M1, the majority of the observations are in favor of melatonin’s anti-inflammatory actions in the regulation of microglial functions. With regard to this overwhelming evidence, an earlier cytokine-based conclusion that melatonin may not be an important modulator of macrophage and microglia function [60] appears to be obsolete. In addition to the many data concerning anti-inflammatory signaling within the microglial regulatory network, various studies have observed the suppression of microglia activation and microgliosis by melatonin under various conditions and often along with attenuated astrocyte activation [61,62,63,64,65,66,67,68,69,70]. Conversely, microglia activation can be aggravated by pinealectomy [71]. Moreover, inflammation upon traumatic brain injury as well as microglia and astrocyte activation has been reported to be suppressed by the synthetic melatonergic agonist, ramelteon [72]. This is in accordance with the recent observation that the inhibition of LPS-induced neuroinflammation by melatonin is mediated by the MT_1_ receptor [73]. This would imply that canonical melatonin receptors are at least required for a substantial part of melatonin’s anti-inflammatory actions on microglia. This does not exclude secondary signaling mechanisms, e.g., via sirtuins and microRNAs [23,74,75]. Contributions from the scavenging of free radicals may also contribute, especially when using strongly elevated melatonin doses.

A detailed listing of pathologies in which anti-inflammatory effects have been observed would be space-consuming and would not reveal novel insights since the protective actions that are now specifically attributable to microglia are largely identical to those known from earlier studies without the specification of cellular cytokine sources. Again, all of the previously known conditions, such as toxicity by LPS and other inflammation-inducing agents, bacterial infections, brain trauma, ischemia/reperfusion, hemorrhage, aging, and neurodegenerative disorders are covered (cf. refs. [2,3,4,21,22]).

## 3. Melatonin Influences Glial and Glial-Neuronal Interactions

### 3.1. Multidirectionality of Interactions

The actions of melatonin on microglia cannot be seen independently of other cells in the CNS. The interplay of neurons, astrocytes, and microglia has been repeatedly addressed in articles on neuroprotection and neurodegeneration as well [76,77,78,79,80], including papers that have focused on melatonin [2,21,81,82]. The multidirectionality of these connections is astonishingly pronounced and important. Although microglia and infiltrating macrophages may appear to represent the major, specialized cell types responsible for inflammation control, astrocytes and neurons are also capable of sending inflammatory signals. All of these cell types express the proteins for inflammasome formation [83] although their subtypes can be cell-specific, such as NLRP1 and AIM2 in neurons, NLRP2 in astrocytes, and NLRP3 in microglia and macrophages [2,84]. Despite this knowledge, literature in the melatonin field has almost exclusively focused on NLRP3. Correspondingly, proinflammatory cytokines, e.g., IL-1β and TNFα, are also released under pathophysiological conditions by neurons [85] and astrocytes [86]. Additionally, neurons may release chemokines such as CCL2 and CXCL5 and proinflammatory peptides such as substance P and CGRP (calcitonin gene related peptide) [85]. Moreover, it should be remembered that astrocytes can secrete cytokines, chemokines, and other factors in the course of developing a senescence-associated secretory phenotype (SASP) [2,87], a process of relevance to low-grade neuroinflammation in neurodegenerative diseases and inflammaging [2,88,89] and is part of the DNA damage response (DDR) [90]. COX-2-dependent prostaglandins are not restricted to microglia and infiltrated macrophages and are also produced and released by astrocytes and neurons [86]. Another important aspect of proinflammatory signaling concerns NO formation. When produced at an elevated level by the nNOS in neurons, NO may activate both astrocytes and microglia, whereas the upregulation of iNOS in astrocytes and, even more so in the microglia or macrophages, has consequences for the other cell types, too, and may result in neuronal overexcitation [21]. The upregulation of eNOS is of importance under ischemia conditions and becomes detrimental when associated with oxidative stress resulting in high peroxynitrite levels [91]. In all of the cells mentioned, and additionally, in the endothelial and other vascular cells, the activation of NADPH oxidases (Nox) [92] leads to enhanced ROS formation, which also stimulates inflammatory responses inasmuch as other causes of oxidative stress do. Independently of cell type, any dying cell can release proinflammatory DAMP factors (damage-associated molecular pattern), such as histone H1 [93] or HMBG1 (high-mobility group box 1) [94,95,96]. Both of them activate microglia, and the suppression of HMBG1 can reduce ROS formation and proinflammatory cytokine release.

### 3.2. Joint Actions of Melatonin against Microgliosis and Astrogliosis

Under various neuroinflammatory conditions, astrocytes and microglia are concomitantly activated [78,97,98,99]. However, it is a puzzling task to discriminate the activations caused in parallel by the same inducer and the interactions between the two cell types [98]. The situation is insofar complicated, as it is not only necessary to consider the polarization subtypes of microglia but to also to distinguish between the corresponding atrocyte subtypes, known as the neurotoxic A1 and the neuroprotective A2 cells [99], a remarkable parallel to microglia and macrophages. Meanwhile, it seems to be certain that microglia can modulate astrocytic properties and activities, but conversely, astrocytes regulate microglia in a corresponding way [98]. The existence of M1 and M2 as well as the A1 and A2 subtypes is highly suggestive for mutual influences driving processes into the same direction.

With regard to melatonin research, the understanding of these crosstalks and the orchestrating role of melatonin are still in their infancy. Along with the multiple documented reductions of proinflammatory cytokines discussed in Section 2.3, the attenuation of both microglia and astrocyte activation has been observed as an obviously general process mediated by melatonin, which can be also followed histochemically. For further details see refs. [36,62,72,78]. Instead of expanding this aspect by enumerating repetitive findings, some more special and sometimes innovative insights shall be discussed.

An influence of melatonin on the infiltration of other cells was reported in rat transient focal cerebral ischemia [100]. It reduced the evasation of neutrophils (Ly6G^+^/CD45^+^) and the infiltration of macrophages/microglia (CD11b^+^/CD45^+^) into the damaged site by about a half. This effect may be caused by several factors, such as the attenuation of cytokine release, including that of CXCL8 (=IL-8). CXCL8 can be released by macrophages and microglia, but it can also be released by various other cell types. Concerning the attraction of macrophages or microglia, the CD11b^+^/CD45^+^ criterion does not yet allow conclusions on polarization [6], but the condition under which these cells are attracted may be in favor of M1.

Several other studies shed light on neuronal/microglial interactions. This became even more evident in primary murine glial cultures and BV2 cells, in which NLRP3 activation and the release of proinflammatory cytokines were shown to depend on the suppression of nAChR (α7 nicotinic receptor), i.e., on the strength of a neuronal signal [33]. Melatonin was shown to attenuate this proinflammatory response, which remained unaffected by melatonin in nAChR knockout cells. Conclusions were extended to an anti-autophagy effect of melatonin [33]. Another neuronal/microglial connection may be deduced from the actions of kainic acid in a study of hippocampal neurodegeneration, in which microglia activation was reduced by melatonin [101]. This finding was interpreted by the authors as a protective action via microglia. Although the effect may be also explained by a primary protection of the kainic acid receptor expressing glutamatergic neurons, it certainly allows the conclusion that neuronal overexcitation has an influence on the microglia, regardless of whether calcium-overloaded astrocytes [102] have been involved, and that melatonin suppresses these interconnected changes.

A recent investigation related neuronal/microglial protection by melatonin to properties of exosomes [103]. In rats subjected to focal ischemic stroke, exosomes from melatonin treated animals were reported to attenuate the pyroptotic death of both the neurons and the microglial cells along with reduction of the infarct size [103]. Plasma exosomes from control and melatonin-treated animals were collected and the protective effects observed with the latter samples indicated that melatonin influences the composition of the exosomal contents towards anti-inflammatory properties. These findings are of high relevance even though the main sources of these exosomes have not been identified. Researchers in the melatonin field should be encouraged to use approaches of this type more often in the future. Other studies concerning the effects of melatonin on exosomes have likewise yielded remarkable insights on the potential of melatonin in modulating this route of intercellular communication, including one on anti-inflammatory effects on macrophages by exosomes from melatonin-treated cells [104]. Moreover, substantial information has meanwhile accumulated on the effects of melatonin on differential microRNA expression, i.e., on molecules of regulatory relevance that represent a variable but also quantitatively and functionally important cargo of exosomes [105,106,107,108].

A rather divergent inflammatory interaction has been described for the relationship between macrophages/microglia and pinealocytes, in which melatonin synthesis itself is affected [109,110]. In pinealocytes, TLR4 activation by PAMP or DAMP factors, such as LPS or Aβ, reduces the rate of melatonin synthesis, but, on the other hand, upregulates TNFR1. However, microglial TLR4 activation causes the formation and the release of TNFα. The binding of TNFα to TNFR1 in pinealocytes re-initiates melatonin synthesis. In this way, the widely NF-κB-mediated inflammatory responses lead to a biphasic dynamics of melatonin secretion, which is first suppressed, but later, in a resolution phase, is resumed.

A further aspect that may gain considerable future importance concerns the role of another still insufficiently considered type of glia, the NG2 cells (Neural-Glial 2 expressing cells, also known as polydendrocytes). These cells, which had previously only been regarded as oligodendrocyte precursors, constitute the fourth large population of glia in addition to microglia, astrocytes, and oligodendrocytes [12]. The NG2 glia is believed to play an important role in the maintenance of microglial homeostasis [14,15], to influence the neuronal/microglial interactions [15], and to participate in energy balance via leptin-sensing neurons [13]. Regarding the latter function, the role of leptin as an nNOS suppressing agent is of interest [111], as this concerns a function shared by melatonin and its metabolite AMK (*N*^1^-acetyl-5-methoxykynuramine) [16,17,18,19,91]. To date, information about the effects of melatonin on NG2 glia has remained rather scarce. The inhibition of NG2 activation by melatonin has been reported [112], but the definite meaning of these changes in a cell type with balancing properties may remain uncertain. In the respective study, a rather powerful insult by methamphetamine toxicity led to NG2 proliferation, which was associated with astrocyte and microglia activation, effects that were attenuated by melatonin [112].

## 4. Melatonin’s Actions via Sirtuins as Related to Microglia

The notion that several protective actions of melatonin are mediated by the upregulation of sirtuins, most particularly, but not exclusively, the upregulation of SIRT1, has opened a new field of melatonin research. The criteria for the involvement of SIRT1 are convincing when effects by melatonin are suppressed by SIRT1 inhibitors such as sirtinol or, more specifically, by EX527, or by downregulation using *Sirt1* siRNA [4,21,23,74,113]. In quantitative terms, this evidence is already overwhelming for nontumor cells, whereas the relationship is the opposite in tumor cells [4,23,74,113]. Importantly, there is a substantial overlap in the action spectra of melatonin and SIRT1 in the field of anti-inflammatory protection [4,23,74,113]. This concerns suppressive actions against the key steps of inflammation induction, such as TLR4 signaling, NLRP3 activation, NF-κB signaling (at the expense of Nrf2 activation), and the expression and release of proinflammatory cytokines as well as HMGB1 release and signaling. Additionally, overlaps exist in mitochondrial protection, aging, and circadian regulation.

Meanwhile, this relationship between melatonin and SIRT1 has become evident in the control of microglia, too. In murine microglia, NLRP3 activation in Iba1^+^-cells (ionized calcium-binding adapter molecule 1) was suppressed by melatonin, an effect abolished by SIRT1 inhibition [32]. Similar findings were obtained in LPS-treated BV2 cells, in which melatonin-dependent upregulation of Nrf2 was inhibited by EX527 [114]. In a hypoxia model, both BV2 cells and rat primary microglia exhibited increased NF-κB translocation to the nucleus, which was SIRT1-dependently inhibited by melatonin [47]. In a rat ischemia model from the same article, melatonin was shown to prevent decreases in SIRT1 expression in amoeboid Iba1^+^ cells of the corpus callosum. Another study on LPS-stimulated BV2 cells reported SIRT1-dependent inhibition of the TLR4/MyD88/NF-κB pathway by melatonin, along with a suppression of HMGB1 release [39]. This latter role of SIRT1 is also plausible with regard to its HMGB1-deacetylating activity since acetylation of this alarmin is required for release [115,116]. In Aβ_42_-treated HMC3 cells, a human microglial cell line, melatonin was recently shown to not only prevent NF-κB nuclear translocation, but to also cause prolonged upregulations of SIRT1 and BDNF (brain-derived neurotrophic factor) [48]. Notably, BDNF is a factor released by M2 microglia [9].

The list of publications referring to the melatonin–SIRT1 nexus in microglia is still relatively short. However, this should be regarded as a starting point for an emerging trend that will gain attention and importance in the near future.

## 5. Melatonin and MicroRNAs: Relevance to Microglia

MicroRNAs have become another hot topic in cell biology. In the context of melatonin and SIRT1 as its downstream factor, several microRNAs have been identified that play a role in inflammation control [23,117]. Among them, the miR-23 cluster, the miR-24 cluster, the miR-155 cluster, and the miR-132/212 cluster, all of which target *Sirt1* mRNA, were downregulated by melatonin, thereby preventing a blockade of SIRT1 expression. Although the expression of these miRNAs is known from macrophages, their investigation in microglia is still in its beginning. In the case of miR-155, its proinflammatory role in the microglia has been confirmed [118,119,120,121]. It was also concluded to favor M1 polarization in microglia, and its suppression was associated with polarization to M2 [121]. miR-132-5p was reported to participate in the activation of microglial TLR7/8 followed by proinflammatory cytokine and chemokine release [122].

The importance of melatonin-modulated microRNAs to microglia may be assumed with regard to the above-mentioned protective changes in exosome composition by melatonin treatment [103]. The described attenuation of pyroptosis in both microglia and neurons strongly indicates that the exosomal contents can convey the suppression of TLR4/NF-κB signaling. The decisive factors may be microRNAs, but it should be underlined that exosomes also contain many other components, including other RNAs, such as lncRNAs (long noncoding RNAs), snoRNAs (small nucleolar RNAs), and circRNAs (circular RNAs), and regulatory proteins. Thus, the observed effects should not be precociously and exclusively attributed to microRNAs. Another intriguing but unclarified point would be that of whether melatonin-induced changes might be primarily caused by modulation of microRNA sponges, such as circRNAs and several lncRNAs. At least in the context of inflammation, as far as it is under circadian control, several circRNAs that sponge inflammation-related microRNAs have been identified [117].

## 6. Melatonin and Microglia Polarization: Urgent Need for Further Consideration

In the melatonin literature, the topic of polarization preferably concerns macrophages [5]. Although many data on iNOS and cytokines obtained in the brain may be interpreted in terms of changes in microglia, polarization of this cell type under the influence of melatonin has been infrequently addressed. Moreover, conclusions are sometimes affected by the relative unspecificities of the markers.

In the visual cortex of mice, inflammation by microelectrode implantation was attenuated by melatonin, which also increased the number of Arg1^+^ (arginase-1^+^) cells. As Arg1 is a reliable marker of M2 microglia [9], a shift toward M2 polarization should have taken place [123]. In a study on spinal cord injury in rats, melatonin increased the number of Arg1^+^/CD206^+^ cells and decreased that of iNOS^+^/CD16^+^ cells [27] using two specific markers of M2 and M1 microglia, respectively [9]. In the reperfusion state after ischemic stroke, melatonin enhanced the rate of TREM2/iNOS [42]. While iNOS certainly reflects an aspect of the M1 phenotype, TREM2 (triggering receptors expressed on myeloid cells-2) is exclusively expressed in the microglia, but there is discordance on the subtype specificity of this marker [124]. Therefore, the conclusion of a polarization shift toward M2 [42] appears to be likely but is affected by some uncertainty. The reduction of M1 polarization by melatonin was also concluded in a study on the roles of prorenin and UCP2 (uncoupling protein-2) in mitochondria, in which prorenin-induced expression of CD86 and TNFα release were counteracted by melatonin [125]. CD86 is often regarded as an M1 marker of microglia [9], but, at least in macrophages, it is also present in the M2b phenotype [7]. Despite this remaining uncertainty, the conclusion is presumably justified. A more detailed investigation was conducted by combining distal middle cerebral artery occlusion in mice with neuron–microglial co-cultures and experiments in BV2 cells [126]. In both the affected brain and BV2 cells, melatonin caused downregulation of M1-typic markers and products (CD11b, CD86, iNOS, IL-1, IL-6, TNFα) and upregulation of the M2-typic ones (CD206, Arg1, TGFβ, IL-10, YM1/2). YM1/2 (=Chi3L3/Chi3L4) are two chitinase-like proteins originally attributed to macrophages and neutrophils, which also appear to be M2 microglia markers [127]). In BV2 cells, melatonin increased the phosphorylation of STAT3, which is known to be a decisive factor toward M2 polarization [126].

**Figure 2:**
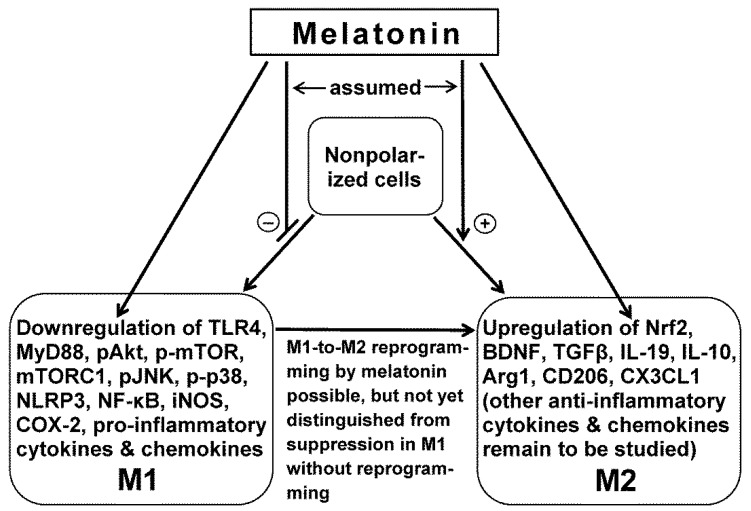
Main effects of melatonin in microglia, as indicated by the majority of studies (deviating findings have been reported for retinal microglia [57]). Melatonin favors polarization to M2, as shown by the upregulation of M2-specific markers, such as Arg1 and CD206, and disfavors M1, as indicated by the downregulation of iNOS. Indirect actions via other cell types are omitted but have been addressed in the current text. Of note: several of melatonin’s actions are mediated by SIRT1 (sirtuin 1; cf. current text). Abbreviations: COX-2, cyclooxygenase-2; iNOS, inducible nitric oxide synthase; mTORC1, mTOR complex 1; MyD88, myeloid differentiation factor 88; NF-κB, nuclear factor κB; NLRP3, nucleotide-binding oligomerization domain, leucine-rich-containing family, pyrin domain-containing-3; Nrf2, nuclear factor erythroid 2-related factor 2; pAkt, phosphorylated Akt kinase; p-mTOR, phosphorylated mechanistic target of rapamycin; p-p38, phosphorylated protein 38. For other abbreviations see the legend of Figure 1 and the current text.

Collectively, these studies are in favor of an anti-inflammatory action of melatonin via microglia M2 polarization (Figure 2). However, the above-mentioned findings on M1 polarization in the model of macular degeneration [57] remain. Whether or not this discrepancy is really controversial will also depend on the fundamental question of multiple the roles of the polarized macrophages, i.e., whether they only play a role in inflammation or if they also have a role in other functions, such as in angiogenesis.

## 7. Conclusions

Most of the information summarized in this article indicates that the effects by melatonin on microglia largely resemble those known for macrophages. They are consistent with numerous findings obtained in studies on the CNS, in which microglia was not specifically addressed although the suppression of TLR4 and NLRP3 activation and downstream factors as well changes in pro- and anti-inflammatory cytokines were the most easily interpretable as effects on microglia.

Nevertheless, a number of uncertainties and gaps have remained at the present state of knowledge. On the one hand, it seems obvious that melatonin favors the M2 polarization of microglia at the expense of M1, in a way that is similar to the one known for macrophages (cf. ref. [5]). However, unequivocal interpretations largely depend on the demonstration of markers with high subtype specificity. Several markers are not specific to microglia but are equally present in macrophages. This may not be relevant as long as the applied methods of inflammation induction are not associated with the infiltration of macrophages and other leukocytes such as neutrophils.

A particular problem exists in the case of iNOS. In fact, iNOS is a marker of M1 but not M2 microglia [9]. However, if the determinations have not been done by multiple labeling, including a microglia-specific marker such as TREM2, and if no M2-specific markers such as Arg1 and CD206 have been included, the mere reduction of iNOS expression in a tissue sample may not indicate that a re-programming from M1 to M2 has occurred. iNOS is also expressed in other cells, such as astrocytes. From a principle point of view, it may be necessary to also distinguish between a change in polarization and the suppression of a function that is typical for one polarization subtype. If we regard the polarization of microglia and macrophages as kind of a differentiation in a developmental process, it may be possible to suppress or silence a gene without fully re-programming the polarization type. There is a good reason for this opinion: iNOS is present in numerous cells, and it is downregulated by melatonin in various tissues. No one would expect that the downregulation of iNOS in the heart, diaphragm, liver, and lung [17,18,128,129] could be a matter of the polarization of the respective nonmyeloid cells in these tissues.

Another gap exists with regard to the subtypes of M2 microglia. As these subforms are involved in a sequence of functions in the course of healing and reorganization after injury, a profound and complete understanding of M2 properties under the influence of melatonin would require differential considerations of the changes in the subtypes. In principle, subtypes can be identified by certain ensembles of markers, such as CD206, CD209, TGFβ, and CCL22 in M2a; CD16, CD32, CD64, and IL-10 in M2b; and CD163 and IL-10 in M2c [6]. Several of these markers have been considered in the studies summarized here, but the proportions between the subtypes remain unknown.

Moreover, attention should be increasingly directed toward the routes of the secondary signaling downstream of melatonin receptors, in particular, by SIRT1 and SIRT1-associated pathways, and to the wide field of noncoding RNAs, microRNAs in particular, but also lncRNAs, circRNAs, and, with regard to melatonin’s chronobiological role, circadian-associated snoRNAs, asRNAs (antisense RNAs), and the various types of enhancer RNAs [130]. Despite the complexity of the RNA world, all of this may turn out to be of utmost relevance to microglia and the regulation of microglia by melatonin. The initial findings on anti-inflammatory regulation by melatonin-programmed exosomes indicate that this direction of experimentation is a highly promising route for future melatonin research.

## Data Availability

Not applicable.
